# Dynamic interactions between *Candida albicans* and different streptococcal species in a multispecies oral biofilm

**DOI:** 10.1002/mbo3.1381

**Published:** 2023-10-03

**Authors:** Tenzin Kunchok Lueyar, Lamprini Karygianni, Thomas Attin, Thomas Thurnheer

**Affiliations:** ^1^ Division of Clinical Oral Microbiology and Immunology, Clinic of Conservative and Preventive Dentistry Center of Dental Medicine, University of Zurich Zurich Switzerland

**Keywords:** *Candida albicans*, CLSM, FISH, multispecies biofilm, oral cavity, streptococci

## Abstract

The oral cavity is colonized by a plethora of bacteria, fungi, and archaea, including streptococci of the mitis group (MSG) and the yeast *Candida albicans*. This study aims to investigate the role of streptococcal species in the development of oral biofilm and the cross‐kingdom interactions between some of the members of the commensal MSG and the pathogen yeast *C. albicans* using a multispecies supragingival biofilm model. A total of nine different in vitro biofilms were grown, quantified with culture analyses, and visually examined with confocal laser scanning microscopy (CLSM). A four‐species biofilm without any streptococcal species was used as a basic biofilm. In each subsequent inoculum, one species of MSG was added and afterward combined with *Streptococcus mutans*. The eight‐species biofilm contained all eight strains used in this study. Culture analyses showed that the presence of *S. mutans* in a four‐species biofilm with *Streptococcus oralis* or *S. oralis* subsp. *tigurinus* did not differ significantly in *C. albicans* colony‐forming unit (CFU) counts compared to biofilms without *S. mutans*. However, compared to other *mitis* species, *Streptococcus gordonii* combined with *S. mutans* resulted in the lowest CFUs of *C. albicans*. Visual observation by CLSM showed that biofilms containing both *S. mutans* and one species of MSG seemed to induce the formation of filamentous form of *C. albicans*. However, when several species of MSG were combined with *S. mutans*, *C. albicans* was again found in its yeast form.

## Graphical abstract

There is evidence that multispecies biofilms containing oral streptococci and the yeast *Candida albicans* may negatively affect the host and even promote infections. The results of this study show the presence of *Streptococcus mutans* in a four‐species biofilm with *Streptococcus oralis* or *S. oralis* subsp. *tigurinus* did not differ significantly in *C. albicans* colony‐forming unit (CFU) counts compared to biofilms without *S. mutans*. However, compared to other *mitis* species, *Streptococcus gordonii* combined with *S. mutans* resulted in the lowest CFUs of *C. albicans*.

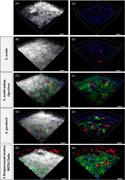

## 1 INTRODUCTION

Infections caused by polymicrobial biofilms are becoming increasingly significant (Diaz et al., 2012; Peters et al., 2012; Souza et al., 2020; Xu, Sobue, et al., 2014; Zhou et al., 2022) and several investigations of these infections have led to a better understanding of how the interactions between species influence the outcome of a disease. An ecosystem as rich and diverse as the oral cavity harbors bacteria, fungi, and archaea (Wade, 2013). Due to their significant association with oral diseases, bacterial–fungal interactions have gained increasing attention and interest. The human oral cavity is colonized by streptococci of the mitis group (MSG) and the yeast *Candida albicans*, among others. There is evidence in the literature that multispecies biofilms containing both microorganisms may negatively affect the host and even promote infections (Förster et al., 2016).

*C. albicans* belongs to the most prevalent and studied fungal species in the human microbiota, is found asymptomatically throughout the body, and colonizes the gastrointestinal and genital–urinary tracts (Poulain, 2015). Due to its ability to change its morphology from yeast to pseudo‐ or hyphal forms (referred to as “filamentous forms” in this study), *C. albicans* can significantly contribute to pathogenicity (Mayer et al., 2013). Through its ability to change its morphology, an advantage is created for the adhesion of *C. albicans* to more sites and niches, resulting in its pathogenic character as hyphae (O'Donnell et al., 2015). Significantly, the co‐adherence of *C. albicans* to oral bacteria is essential for it to colonize and survive in the oral cavity (Liu et al., 2020).

Streptococci are localized throughout the human body and comprise the dominant species in the saliva, upper respiratory tract, and oral cavity (Abranches et al., 2018). Streptococcal species are differentiated based on their type of hemolysis on blood agar plates (Sherman, 1937). In the past, oral streptococci were known as *viridans* streptococci (Abranches et al., 2018). However, with the constant discovery of new streptococcal species, it became evident that additional criteria were needed to distinguish between the increasing number of streptococcal species. DNA and 16S ribosomal RNA (rRNA) sequencing facilitate more accurate species‐level bacterial identification and an enhanced understanding of phylogenetic relationships (Kawamura et al., 1995). Initially, oral streptococci were classified into four groups (Whiley & Beighton, 1998), but in a new classification proposed by Richards et al. (2014), a more robust phylogenetic approach yielded eight distinct groups: *mitis*, *sanguinis*, *anginosus*, *salivarius, downei*, *mutans*, *pyogenic*, and *bovis*. To date, all groups except the pyogenic and bovis groups contain oral streptococci. Regarding the oral cavity, the MSG is the largest group, which currently comprises 20 species. Distinguishing species within the MSG (especially *Streptococcus oralis* and *Streptococcus mitis*) based on the 16S rRNA method alone can be very challenging. In particular, MSG are the first organisms found in the mouths of newborns and are considered primary colonizers, enabling the development of complex microbial communities in the oral cavity (Abranches et al., 2018).

Gram‐positive bacteria such as *Streptococcus mutans* are considered to be the main cariogenic microorganisms to inhabit the oral cavity and form multispecies biofilms known as dental plaque (Zero et al., 2009). Their ability to produce large quantities of acids and glucans allows them to circumvent the salivary buffering capacity. The decreasing pH and the ability to grow at low pH give streptococci an advantage over noncariogenic bacteria (Lemos et al., 2013). In this context, a key factor of streptococcal pathogenicity arises from their ability to survive in the acidic oral environment via modulation of sugar metabolism and irreversible binding to the surface of the tooth. Co‐aggregation with other microorganisms is the second step of biofilm growth and its expansion to other areas of the oral mucosa (Metwalli et al., 2013).

Although *S. mutans* has long been considered the etiological microorganism of dental caries, recent investigations indicate an increased prevalence of *S. mutans* in oral biofilms at sites where *C. albicans* is also present. This suggests that the relationship between these two diverse species may be part of the complex mechanisms involved in the development of caries (Barbieri et al., 2007; Jarosz et al., 2009). Such interactions were also revealed in several clinical studies of early childhood caries (ECC), which is an aggressive form of dental caries affecting children (Hajishengallis et al., 2017). In addition to the main *S. mutans* infection, plaque biofilms from toddlers with ECC usually contain large levels of the fungus *C. albicans* (de Carvalho et al., 2006; Raja et al., 2010; Yang et al., 2012). But the biofilm matrix is thereby identified as a key factor in the pathogenesis of dental caries, since for instance a high‐sugar diet strongly encourages this disease. Namely, in the presence of sucrose, a symbiotic relationship between *C. albicans* and *S. mutans* is mediated through the action of glucosyltransferases (Gtfs) exoenzymes, specifically glucosyltransferase B (GtfB). Even in hyphal form, GtfB attaches to the surface of *C. albicans* cells (Hwang et al., 2015), creating a significant amount of glucans on the *Candida* surface (Falsetta et al., 2014; Gregoire et al., 2011). Insoluble and soluble glucans are the main components of extracellular polysaccharides (EPS) and are essential for forming the core of the biofilm matrix (Bowen & Koo, 2011). Furthermore, *S. mutans* Gtfs expression is induced by the presence of *C. albicans* in mixed‐species biofilms. The matrix facilitates accumulation and adherence to the tooth surface, thus increasing the virulence of *S. mutans* and *C. albicans* for its host (Falsetta et al., 2014).

Comprising 20 species, some members of MSG such as *S. oralis, Streptococcus gordonii, S. mitis, and Streptococcus sanguinis* belong to the most common and commensal oral colonizers in humans (Abranches et al., 2018; Zheng et al., 2016). These streptococcal species are also found in the gastrointestinal and the female genital tracts, and, on entering the bloodstream, they can cause infections (Spellerberg & Brandt, 2011). For instance, one of the novel subspecies of *S. oralis*, the Gram‐positive *S. oralis* subsp. *tigurinus* (former *S. tigurinus*) was shown to play a key role in various infections such as infective endocarditis (Zbinden et al., 2015). Zbinden et al. (2014) detected *S. oralis* subsp. *tigurinus* in saliva and subgingival plaque samples. Due to the difficulties in identifying bacterial species of MSG and the impossibility of distinguishing between *S. oralis* and *S. oralis* subsp. tigurinus using conventional methods, it can be assumed that the prevalence and role of *S. oralis* subsp. *tigurinus* is underestimated in oral infections (Zbinden et al., 2015).

Several studies demonstrated that *C. albicans* as yeast cells enhance and support *S. mutans* adherence to dental surfaces (Jarosz et al., 2009; Raja et al., 2010). Furthermore, scanning electron micrographs show co‐aggregation of *C. albicans* and *S. mutans* in multispecies biofilm grown on human teeth and hydroxyapatite (HA), which indicated a high affinity of *S. mutans* for the hyphae of *C. albicans*. Using in vivo experiments, Klinke et al. (2011) provided evidence of the fungal potential to induce dental caries when present in high counts. The data from another study in which *C. albicans* positively correlated with caries in children confirmed the role of *C. albicans* in the development and progression of dental caries (Raja et al., 2010). Further studies showed that the symbiotic relationship between *C. albicans* and oral streptococci can cause increased fungal (Xu, Sobue, et al., 2014) or bacterial (Falsetta et al., 2014) pathogenicity (Diaz et al., 2012). For instance, the inflammatory response was more pathogenic in a co‐infection of *C. albicans* and *S. oralis* compared with monotypic infections (Xu, Sobue, et al., 2014), and the virulence increased as a result of *C. albicans* facilitating the invasion into the oral mucosa (Diaz et al., 2012).

Since it is essential to learn more about the effects of each species in the biofilm, especially concerning the bacterial–fungal relationship, this study aimed to investigate the interactions between members of the streptococcal group and *C. albicans*, specifically in terms of the cell morphology of *C. albicans*. To this end, a “supragingival” biofilm model was used in the present study. The microbial morphology was analyzed visually using confocal laser scanning microscopy (CLSM), while oral bacterial species and *C. albicans* were quantified using culture analyses. To our knowledge, this is the first time that interactions between *C. albicans* and S*. oralis* subsp*. tigurinus* were described in an eight‐species biofilm model.

## 2 MATERIALS AND METHODS

### 2.1 Formation of multispecies supragingival biofilms

**Figure 1:**
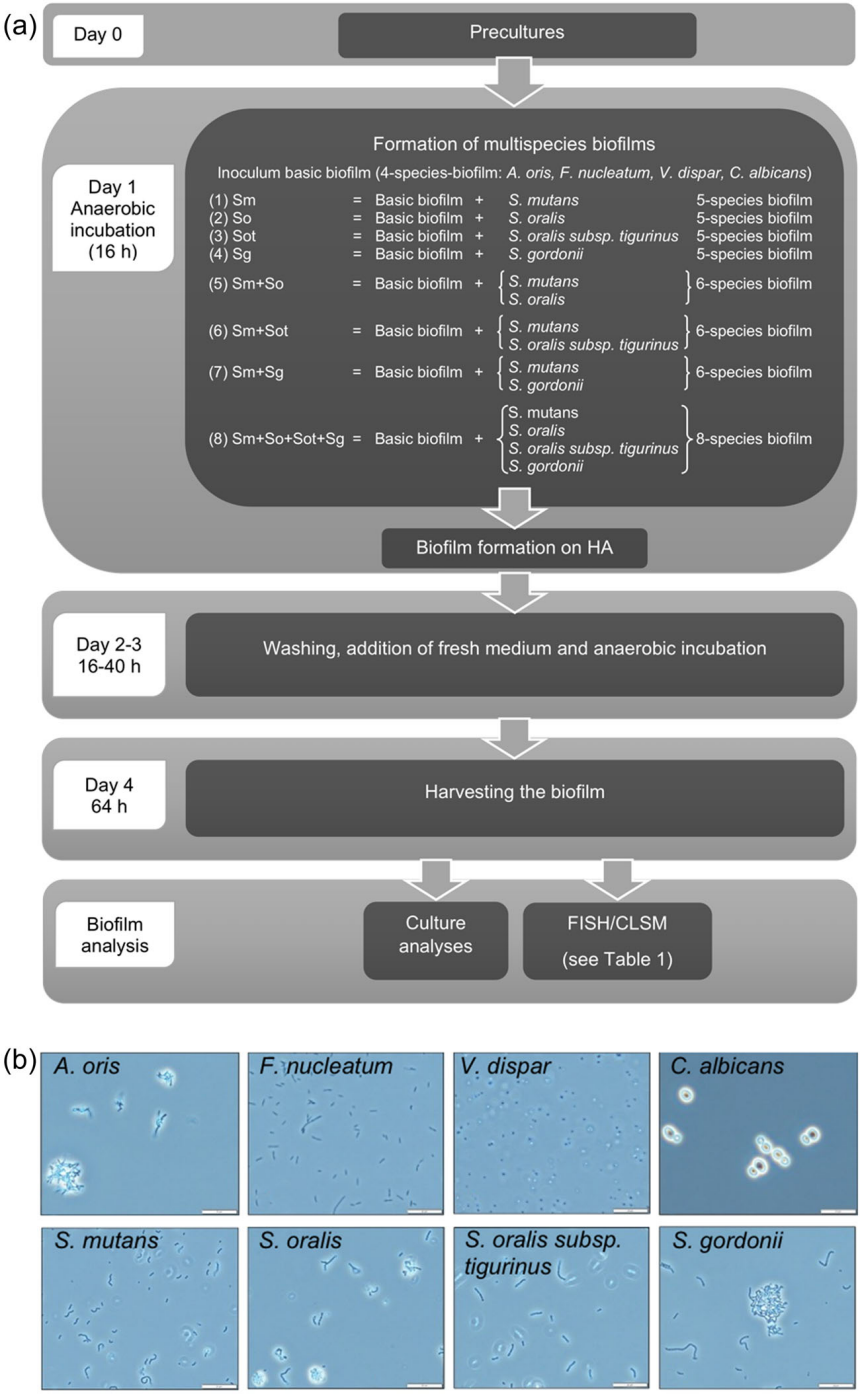
Biofilm formation and analysis using culture and fluorescence in situ hybridization/confocal laser scanning microscopy (CLSM) (a) and microscopic control of the strains (b). Phase‐contrast images of the microbial suspensions of the biofilm strains were taken with an Olympus BX61 with the ×100 Oil 1.35 numerical aperture objective. FISH, fluorescence in situ hybridization; HA, hydroxyapatite.

*C. albicans*, strain ATCC 32032^T^ (OMZ 1134); *S. mutans*, strain ATCC 700610 (OMZ 918); *S. oralis*, strain OMZ 607 SK248; *S. oralis* subsp. *tigurinus*, strain CCOS 600^T^ (OMZ 1133); *S. gordonii*, strain ATCC 10558^T^ (OMZ 505); *Veillonella dispar*, strain ATCC 17748^T^ (OMZ 493); *Fusobacterium nucleatum*, strain OMZ 598; and *Actinomyces oris*, strain OMZ 745 were used in the current study (Figure 1). For the precultures, all strains were transferred onto blood agar plates (Columbia blood agar, CBA, supplemented with 5% whole human blood) and incubated anaerobically at 37°C for 72 h, except for *C. albicans*, which was cultivated with 10% CO_2_ at 37°C. After transferring the strains into modified fluid universal medium (mFUM) (Gmür & Guggenheim, 1983) containing 0.3% glucose (with an additional 1% sodium lactate for *V. dispar*) and overnight incubation, the strains were transferred to fresh mFUM medium and stored anaerobically (except *C. albicans*: 10% CO_2_) for a maximum of 7 h at 37°C. The development and contamination were visually and microscopically examined and all the strains were kept on ice during processing.

For the inoculum, each microbial suspension was adjusted to a defined optical density (OD) at 550 nm (OD_550_ = 1.0) and mixed in equal volumes (1 mL). For the culture analysis of the different bacteria, 50 µL of each dilution was plated using a spiral plater (Eddy Jet; IUL Instruments) onto CBA solid media (BD Difco CBA Base) supplemented with 5% human blood, while for the culture analysis of *C. albicans*, 50 µL was plated onto BIGGY solid media (BD BBL BIGGY agar). After an incubation of 72 h at 37°C in an anaerobic atmosphere (5% CO_2_, 10% H_2_, 85%N_2_) and in 10% CO_2_ (BIGGY), the colony‐forming units (CFUs) were counted with the help of a stereomicroscope.

Pasteurized whole unstimulated pooled human saliva (termed saliva in the following) was collected from donors of the Division of Clinical Oral Microbiology and Immunology at the University of Zurich, with their consent, and incubated at room temperature for 4 h while being gently shaken at 95 rpm to enable pellicle formation on HA discs (Sintered HA Disc, Ø 9 mm; Clarkson Chromatography Products Inc.). The exact procedure for the collection and processing of the saliva was described elsewhere (Guggenheim, Giertsen, et al., 2001). Before the biofilm formation, all HA discs were conditioned with a processed human saliva pool for at least 4 h at room temperature while being gently shaken at 95 rpm. This step aimed to build up a pellicle on the surface due to the importance of the protein interactions with the microorganism and the attachment to the surface. After pellicle formation, HA discs were transferred to 24‐well culture dishes containing 1120 μL processed saliva with 480 μL medium 1 (mFUM + 0.3% glucose) per well, which was anaerobically equilibrated for 45 min at 37°C to reduce the media.

To start the experiment, 200 μL of the inoculum (containing equal volumes of each bacterial suspension) was added to the previously prepared and equilibrated wells. The cell culture plates were subsequently incubated for 16 h at 37°C under anaerobic conditions to achieve the initial biofilm formation on the HA discs. All discs were dip‐washed three times per day and the culture media was refreshed every morning. Supplemented mFUM with 0.3% glucose was used for the first 16 h, followed by an mFUM with 0.15% glucose and 0.15% sucrose for the next 48 h.

### 2.2 Quantification of biofilm species

To harvest the biofilms, the HA discs were vortexed for 1 min in 1 mL of 0.9% NaCl. To ensure that all biofilm had been removed, the surface of the HA discs was randomly examined using CLSM. To ensure the dispersion of the harvested biofilms, they were sonicated for 5 s (level 4/30 W). The bacterial suspensions were then serially diluted in 0.9% NaCl and plated onto agar plates with a spiral diluter (50 μL aliquots). The agar plates were incubated, either anaerobically for CBA or aerobically in the presence of 10% CO_2_ for the BIGGY agar plates, at 37°C for 72 h. The CBA plates were used for the determination of the total CFU count and the CFUs of MSG and *S. mutans*. BIGGY agar plates were used to enumerate the CFUs of *C. albicans*. Species identification was achieved by differentiating the colony morphology and the MSGs were recognized by the green degradation product resulting from their α‐hemolytic activity by light microscopy. The distinction between MSG and *S. mutans* was facilitated by the fact that the *S. mutans* strain used in this study displays no hemolysis.

### 2.3 Fluorescence in situ hybridization (FISH)

To fix the biofilms, the HA discs were incubated in 4% paraformaldehyde for 72 h. To permeabilize Gram‐positive cells, biofilms were pretreated for 15 min in lysozyme (70,000 U/mg) solution with a concentration of 1 mg/mL, followed by an additional soak in mutanolysine (25 U/mL) solution for 4 min. The samples were subsequently stained using multiplex FISH following the slightly modified protocol described earlier (Thurnheer et al., 2004). In brief, before hybridization, the biofilms were preincubated in the dark for 15 min at 46°C in hybridization buffer (0.9 M NaCl, 20 mM Tris‐HCl, pH 7.5, 0.01% sodium dodecyl sulfate [SDS], 25% formamide) without probes. Afterward, the biofilms were immersed in the dark for 5 h at 46°C in the same hybridization buffer additionally containing the 16S rRNA probes MUT590 and MIT447 as well as the 18S rRNA probe Caal1249. The concentrations, sequences, and labels of the probes used in this investigation are listed in Table 1. After hybridization, the biofilms were immersed for 45 min at 48°C in washing buffer (20 mM Tris‐HCl, pH 7.5, 5 mM EDTA, 0.01% SDS, and 159 mM NaCl) and rinsed briefly in physiological saline. The total DNA was stained with 50 µM/mL SYTO40 (Thermo Fisher Scientific) in Nanopure water for 30 min in the dark at room temperature. After staining, each disc was embedded upside‐down on chamber slides (LabTek Chamber Coverglass System) in 50 μL of Mowiol mounting media (Guggenheim, Shapiro, et al., 2001).

**Table 1:** Overview of the probes used in this study.

Probes	Probe concentration (ng/µL)	Fluorescent marker	Formamide (%)	Sequence (5′–3′)	Target organisms
MUT590	10	Cy5	25	ACTCCAGACTTTCCTGAC	*Streptococcus mutans*
MIT447	20	FAM	25	CACYCGTTCTTCTCTTACA	Mitis group
Caal1249	5	CY3	25	GCCAAGGCTTATACTCGCT	*Candida albicans*

### 2.4 CLSM and image analysis

The stained biofilms were analyzed using a CLSM (Leica SP5) with a ×63 oil immersion objective (numerical aperture 1.4). An ultraviolet laser (405 nm excitation), an argon laser (488 nm), a diode‐pumped solid‐state laser diode laser (561 nm), and a helium–neon laser (633 nm) were applied, and the absorption bandwidth was set to 420–470 nm for SYTO40, 490–540 nm for FAM, 570–625 nm for Cy3, and 640–720 nm for Cy5. The scanning of the biofilms was performed in sequential mode to avoid the overlapping between channels and optical sectioning of 0.21 μm was set. The images were acquired in a five‐line average mode and the data were processed with Imaris 9.6.0 (Bitplane AG).

### 2.5 Statistical analysis

Three individual experiments were performed, and each group was represented by triplicate biofilms per experiment. For the statistical analysis, a two‐way analysis of variance was applied to evaluate the differences between each experimental group, followed by Tukey's multiple comparisons test. To allow for logarithmic transformation, all missing values were imputed as the lowest detection limit value of the assay. The significance level was set at *p* < 0.05. All statistical analyses were performed with GraphPad Prism (version 9.0; GraphPad).

## 3 RESULTS

### 3.1 The incorporation of *S. mutans* and MSG members has an impact on *C. albicans* CFUs

**Figure 2:**
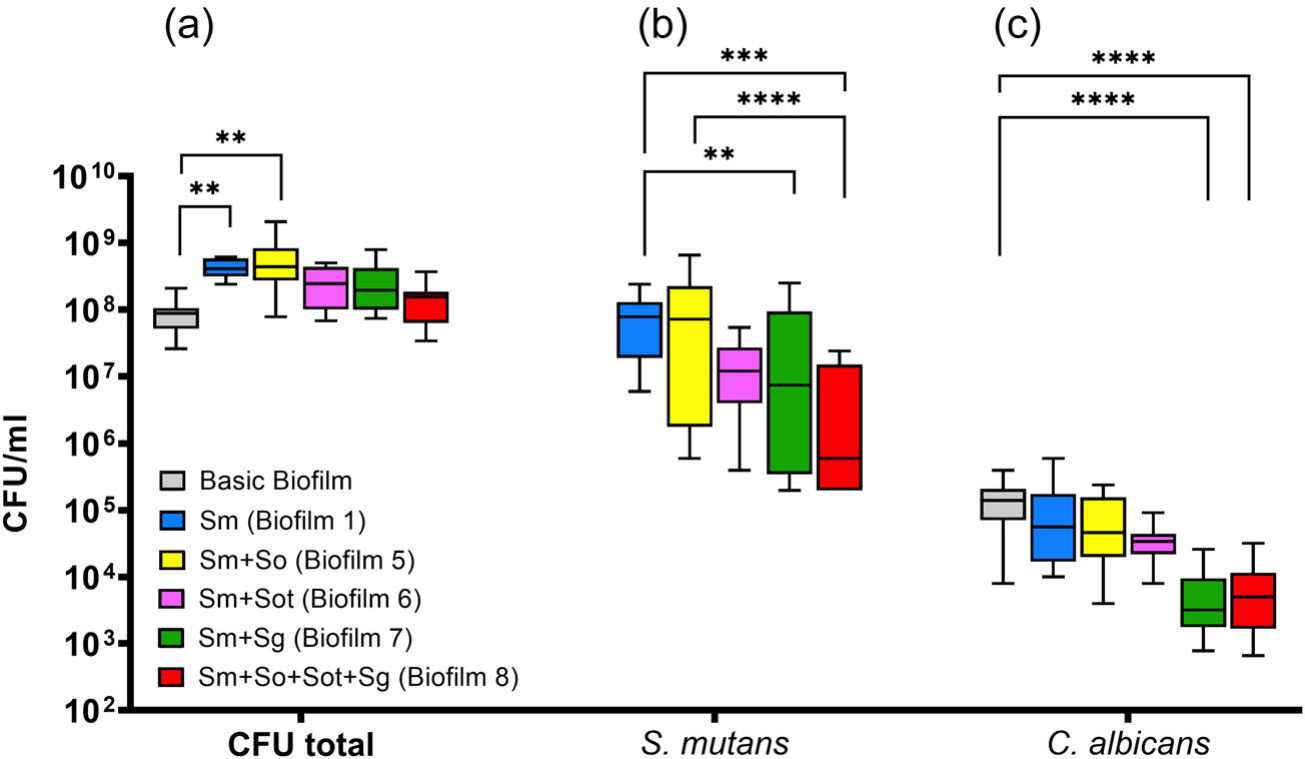
Box plots illustrate the colony‐forming units (CFUs) in interactions between *Streptococcus mutans* and *Candida albicans* in multispecies biofilms. (a) total CFUs, (b) *S. mutans* CFUs, and (c) *C. albicans* CFUs. The different colors indicate the different biofilms and, read from left to right (in each panel), represent the basic biofilm (gray), basic biofilm with *S. mutans* (biofilm 1; blue), biofilm 1 with *Streptococcus oralis* (biofilm 5; yellow), or biofilm 1 with *S. oralis* subsp. *tigurinus* (biofilm 6; pink), or biofilm 1 with *Streptococcus gordonii* (biofilm 7; green) and the eight‐species biofilm (biofilm 8; red). The box plots show the data from three independent experiments, with each group represented in triplicate biofilm cultures, whereas the CFUs were determined by selective agar plating. The horizontal lines indicate their median values. Statistically significant differences between the boxes are indicated with asterisks (**p < 0.01; ***p < 0.001; ****p < 0.0001).

**Figure 3:**
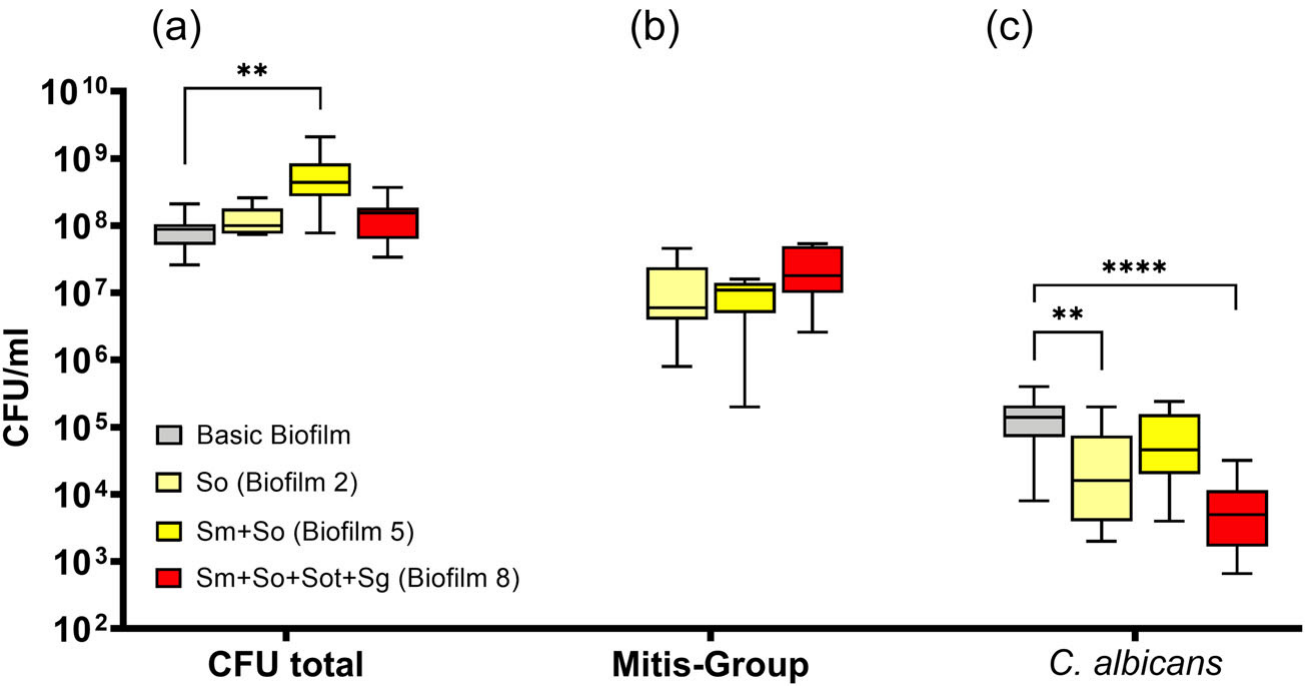
Box plots illustrate the colony‐forming units (CFUs) in the interactions between mitis group streptococci (MGS) focusing on *Streptococcus oralis* and *Candida albicans* in multispecies biofilms. (a) Total CFUs, (b) MGS CFUs, and (c) *C. albicans* CFUs. The different colors indicate the different groups and, read from left to right (in each panel), represent the basic biofilm (gray), basic biofilm with *S. oralis* (biofilm 2; light yellow), biofilm 2 with *Streptococcus mutans* (biofilm 5, corresponding to the six‐species Zürich “supragingival” biofilm model; yellow), and the eight‐species biofilm (biofilm 8; red). The box plots show the data from three independent experiments, with each group represented in triplicate biofilm cultures, whereas the CFUs were determined by selective agar plating. The horizontal lines indicate their median values. Statistically significant differences between the boxes are indicated with asterisks (**p < 0.01; ****p < 0.0001).

**Figure 4:**
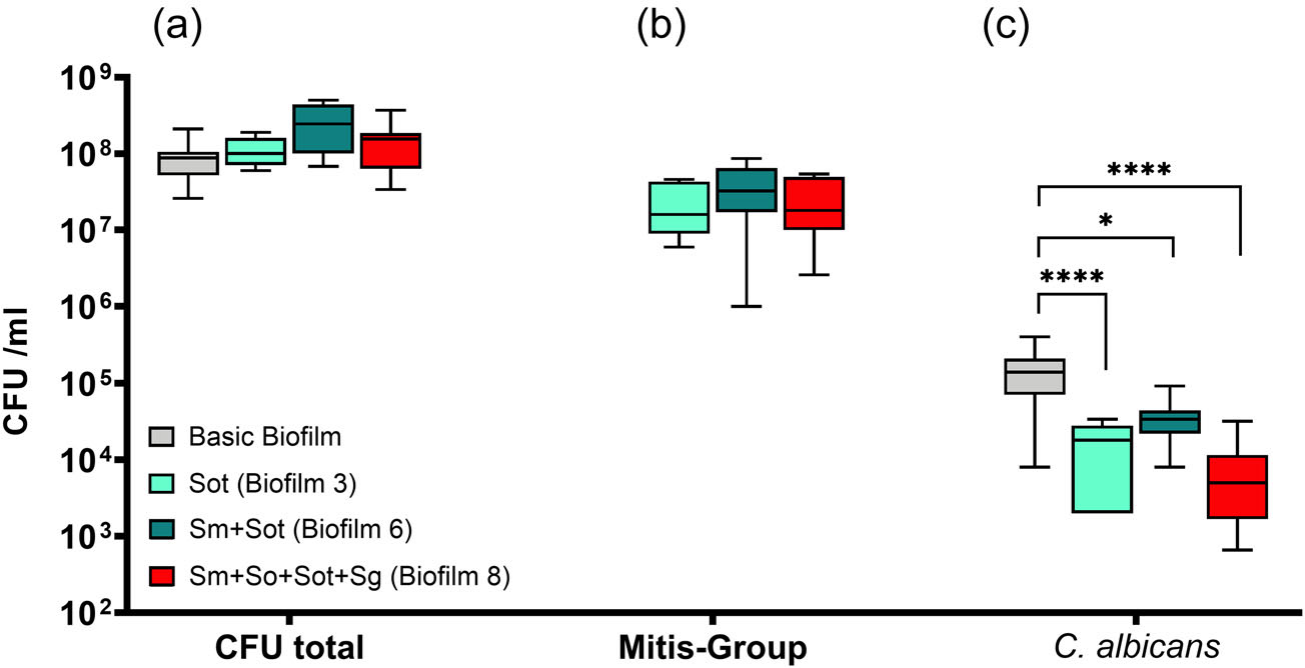
Box plots illustrate the colony‐forming units (CFUs) in interactions between mitis group streptococci (MGS) focusing on *Streptococcus oralis* subsp. *tigurinus* and *Candida albicans* in multispecies biofilms. (a) total CFUs, (b) MGS CFUs, and (c) *C. albicans* CFUs. The different colors indicate the different groups and, read from left to right (in each panel), represent the basic biofilm (gray), basic biofilm with *S. oralis* subsp. *tigurinus* (biofilm 3; light green), biofilm 3 with *S. mutans* (biofilm 6; green), and the eight‐species biofilm (biofilm 8; red). The box plots show the data from three independent experiments, with each group represented in triplicate biofilm cultures, whereas the CFUs were determined by selective agar plating. The horizontal lines indicate their median values. Statistically significant differences between the boxes are indicated with asterisks (*p < 0.05; ****p < 0.0001).

**Figure 5:**
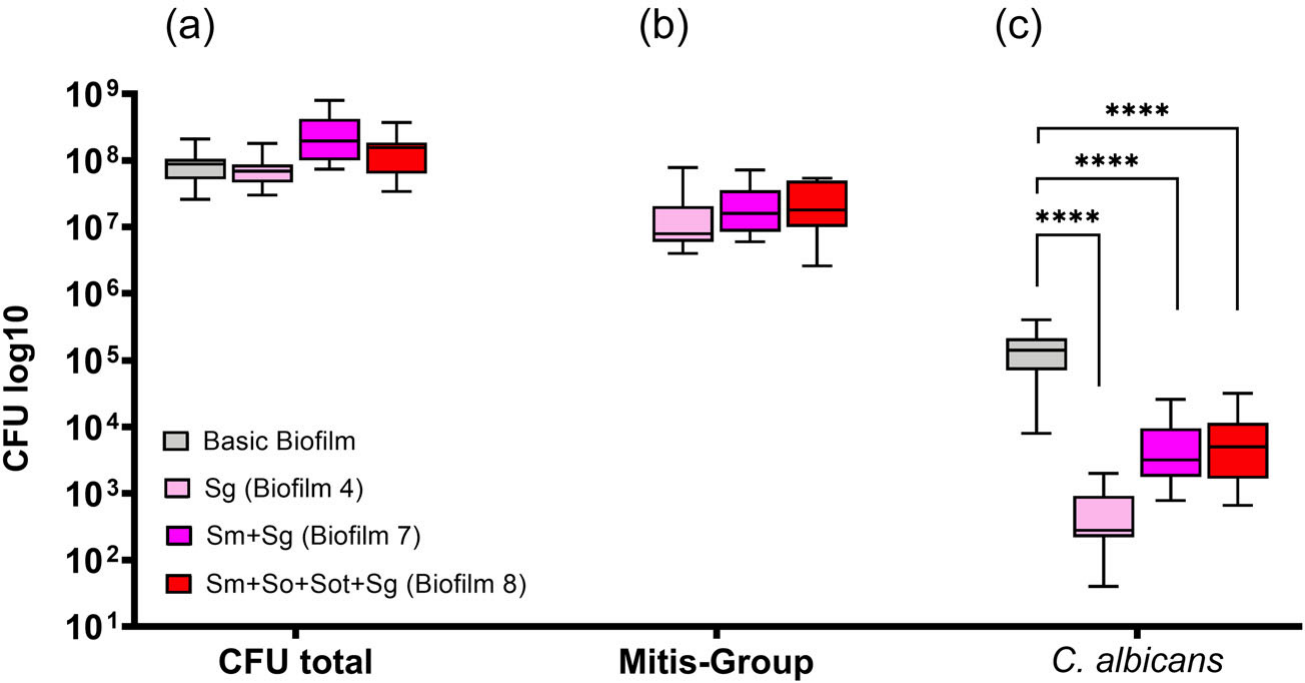
Box plots illustrate colony‐forming units (CFUs) in the interactions between mitis group streptococci (MGS) focusing on *Streptococcus gordonii* and *Candida albicans* in multispecies biofilms. (a) Total CFUs, (b) MGS CFUs, and (c) *C. albicans* CFUs. The different colors indicate the different groups and, read from left to right (in each panel), represent the basic biofilm (gray), basic biofilm with *S. gordonii* (biofilm 4; light pink), biofilm 4 with *S. mutans* (biofilm 7; pink), and the eight‐species biofilm (biofilm 8; red). The box plots show the data from three independent experiments, with each group represented in triplicate biofilm cultures, whereas the CFUs were determined by selective agar plating. The horizontal lines indicate their median values. Statistically significant differences between the boxes shown are indicated with asterisks (****p < 0.0001).

The results showed that the CFU counts of *C. albicans* were lower than that of any bacterial species. A general trend was found in that the counts of *C. albicans* decreased by adding up to seven bacterial species compared to the basic biofilm (Figures 2, 3, 4, 5). Interestingly, biofilms containing both *S. gordonii* and *S. mutans* (Figure 5) showed a significant increase in CFU (*p* < 0.0001; not shown in Figure 5) compared to biofilm 4, which only contained *S. gordonii* in addition to the basic biofilm components.

#### 3.1.1 Impact of different biofilm compositions on *S. mutans*, *C. albicans*, and total CFU counts

First, the impact of the incorporation of *S. mutans* in the basic biofilm consisting of *A. oris, C. albicans, F. nucleatum*, and *V. dispar* on the total CFUs and the CFUs of the mutans group and MSGs were considered (Figure 2). Regarding the total CFUs, only the basic biofilm in the presence of *S. mutans* (biofilm 1) and the basic biofilm combined with *S. mutans* and *S. oralis* (biofilm 5) caused a significant increase (*p* < 0.01) compared to the basic biofilm (Figure 2a).

Regarding the CFU counts of *S. mutans*, the addition of *S. oralis* and *S. oralis* subsp. tigurinus to biofilm 1 (basic biofilm with *S. mutans*) did not yield a significant difference in *S. mutans* CFUs. However, a difference in the presence/absence of *S. gordonii* was evident (Figure 2b). The addition of *S. gordonii* (biofilm 7) to biofilm 1 led to a significant (*p* < 0.01) reduction in *S. mutans* CFU counts compared to biofilm 1 (basic biofilm with *S. mutans*). The reduction of *S. mutans* CFUs was even more significant in the eight‐species biofilm (biofilm 8; *p* < 0.0001) compared to biofilm 1 and also when compared to biofilm 5 (basic biofilm with *S. mutans* and *S. oralis*).

In the case of the CFU counts of *C. albicans*, the addition of *S. mutans, S. oralis*, and *S. oralis* subsp. *tigurinus* to the basic biofilm did not display a significant difference in *C. albicans* CFUs. However, as seen in the *S. mutans* CFUs, the presence of *S. gordonii* (biofilm 7) resulted in significant changes in *C. albicans* CFU counts when compared to the basic biofilm (Figure 2c) and, regardless of whether *S. gordonii* was added alone (biofilm 7) or with *S. oralis* and *S. oralis* subsp. tigurinus together (8‐species biofilm), a significant reduction (*p* < 0.0001, each) in *C. albicans* CFUs was observed compared to the basic biofilm. On the other hand, the addition of *S. mutans* (biofilm 1), *S. oralis* (biofilm 5), or *S. oralis* subsp. *tigurinus* (biofilm 6) to the basic biofilm or biofilm 1, respectively, did not result in significant changes in the *C. albicans* CFU counts.

#### 3.1.2 Impact of different biofilm compositions on the *S. oralis*, *C. albicans*, and total CFU counts

Regarding the total CFU counts of biofilms containing *S. oralis*, the highest numbers were found in the basic biofilm containing *S. mutans* and *S. oralis* (biofilm 2), which corresponds to the standard six‐species Zürich “supragingival” biofilm model (Figure 3). The observed increase in total CFUs of the Zürich biofilm model compared to the basic biofilm was significant (*p* < 0.01). However, neither the biofilm with *S. oralis* but without *S. mutans* (biofilm 2) nor the eight‐species biofilm (biofilm 8) showed significant differences in total CFU counts compared to the basic biofilm (Figure 3a).

Concerning the CFUs of *S. oralis*, neither the addition of *S. mutans* (biofilm 5) nor *S. mutans*, *S. oralis* subsp. *tigurinus*, and *S. gordonii* (eight‐species biofilm, biofilm 8) resulted in a significant difference in MSG CFUs (Figure 3b).

In contrast, the *C. albicans* CFUs were significantly reduced by the addition of *S. oralis* (biofilm 2; *p* < 0.01) and even more significantly (*p* < 0.0001) by the addition of *S. mutans*, *S. oralis* subsp. *tigurinus*, and *S. gordonii* (eight‐species biofilm, biofilm 8) when compared to the basic biofilm. Interestingly, biofilms containing both *S. mutans* and *S. oralis* (biofilm 5) had no impact on the *C. albicans* CFUs (Figure 3c).

#### 3.1.3 Impact of different biofilm compositions on the *S. oralis* subsp. *tigurinus*, *C. albicans*, and total CFU counts

Figure 4 indicates that the presence of *S. oralis* subsp. *tigurinus* does not have a significant effect on total CFUs (Figure 4a) or the CFUs of the MSG (Figure 4b). However, *S. oralis* subsp. *tigurinus* (biofilm 3) significantly reduced the *C. albicans* CFUs (*p* < 0.0001) compared to the basic biofilm, whereas the presence of *S. mutans* with *S. oralis* subsp. *tigurinus* in the basic biofilm (biofilm 6) showed a significant difference (*p* < 0.05) in terms of the *C. albicans* CFUs compared to the basic biofilm. Again, the eight‐species biofilm (biofilm 8) showed a strong and significant CFU reduction (*p* < 0.0001) of *C. albicans* (Figure 4c).

#### 3.1.4 Impact of different biofilm compositions on the *S. gordonii*, *C. albicans*, and total CFU counts

Figure 5 shows that the presence of *S. gordonii* does not have a significant effect on the total CFUs (Figure 5a) or the CFUs of the MSG (Figure 5b). However, regarding *C. albicans*, there is a highly significant difference between the *S. gordonii* (biofilm 4), the biofilm containing *S. mutans* and *S. gordonii* (biofilm 7), and the eight‐species biofilm (biofilm 8; *p* < 0.0001, each; Figure 5c).

#### 3.1.5 Impact of oral streptococci on the biofilm structure and morphology of *C. albicans*

**Figure 6:**
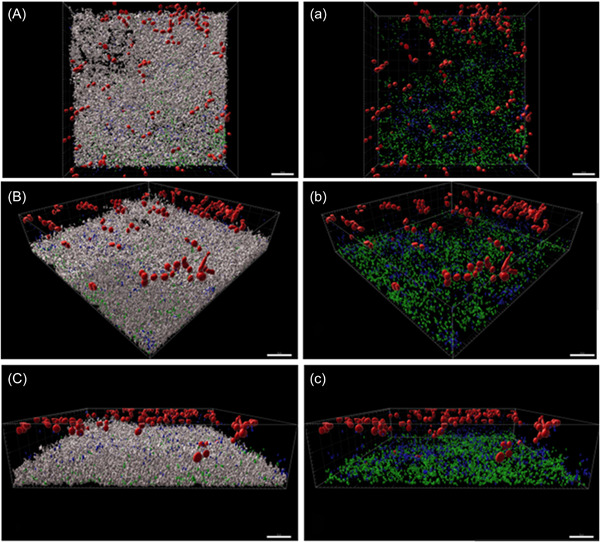
Confocal laser scanning microscopy (CLSM) images exhibiting three‐dimensional (3D) projection representations of the eight‐species biofilm focusing on the biofilm structure. The biofilms were stained with fluorescence in situ hybridization (FISH) at 25% formamide; *Candida albicans* (red), *Streptococcus mutans* (blue), and *Streptococcus oralis*, *S. oralis* subsp. *tigurinus*, and *Streptococcus gordonii* (green). In (A–C), the total biofilm mass is visualized with SYTO40 (light gray) compared to (a–c), where only *C. albicans*, *S. mutans*, *S. oralis*, *S. oralis* subsp. *tigurinus*, and *Streptococcus gordonii* are visualized. To portray the spatial distributions of *C. albicans*, *S. mutans*, and *S. oralis*, *S. oralis* subsp. *tigurinus*, and *S. gordonii* in the biofilm, the same section of the biofilm is shown from different perspectives (Aa, Bb, Cc). The biofilm base in the three‐dimensional reconstructions is downward and the growth direction is upward; scale bar: 20 μm.

By using CLSM the biofilm structure was visually analyzed in this study. The vertical growth was enhanced in the presence of *S. mutans* compared to the biofilms without *S. mutans*, although these visual observations cannot be generalized. The MSG streptococci grew near the salivary pellicle on the HA disc, whereas *C. albicans* was colonizing the biofilm in the middle and upper area as shown in Figure 6.

**Figure 7:**
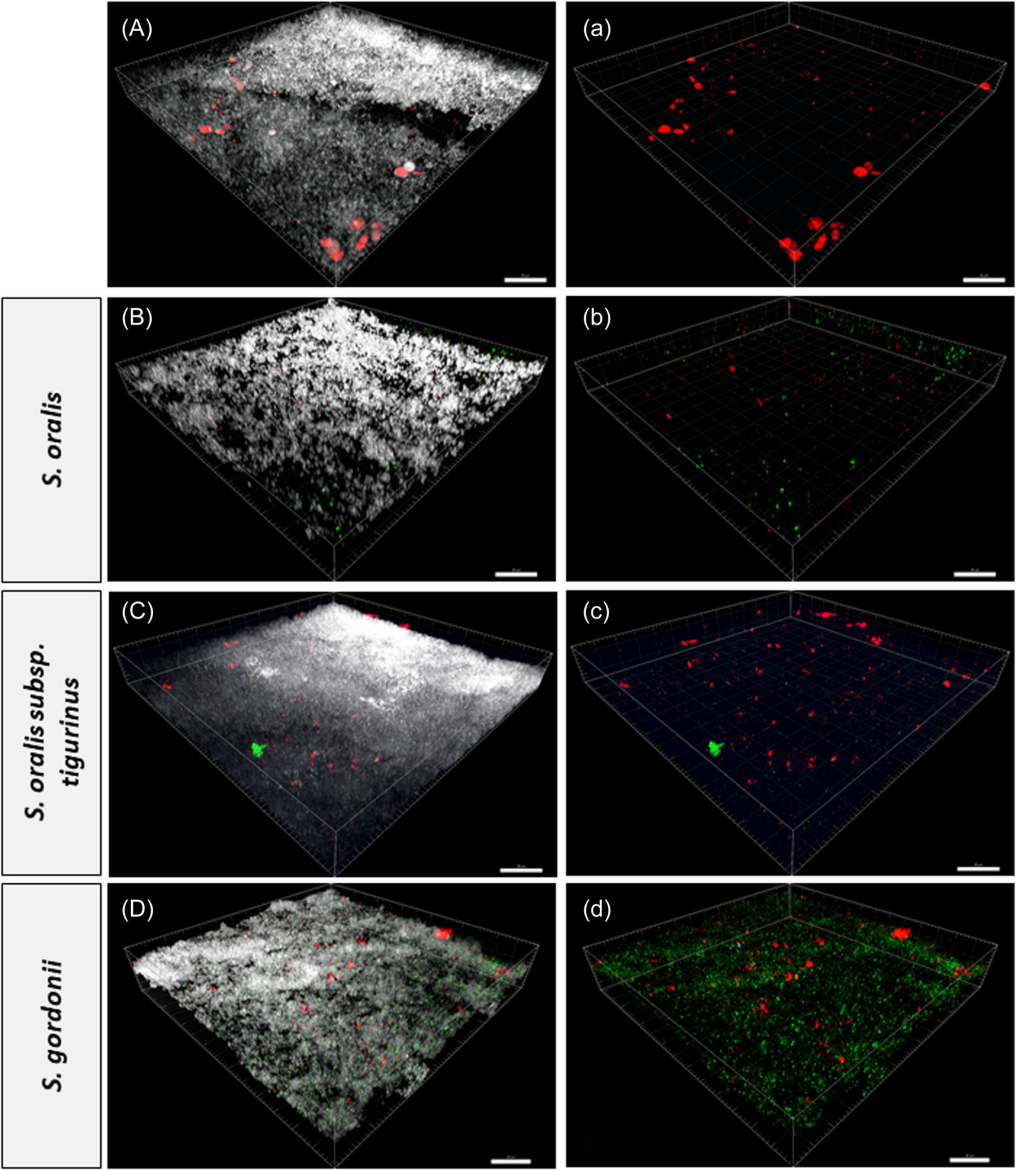
Confocal laser scanning microscopy (CLSM) images exhibiting three‐dimensional (3D) projection representations of the biofilm without *Streptococcus mutans*. The biofilms were stained with fluorescence in situ hybridization at 25% formamide; *Candida albicans* (red), *Streptococcus oralis*, *S. oralis* subsp. *tigurinus*, and *Streptococcus gordonii* (green). In (A–D) the total biofilm mass is visualized with SYTO40 (light gray) compared to (a–d), where only *C. albicans, S. oralis*, *S. oralis* subsp. *tigurinus*, and *S. gordonii* are visualized. The biofilm base in the 3D reconstructions is downward and the growth direction is upward; scale bar: 20 μm.

**Figure 8:**
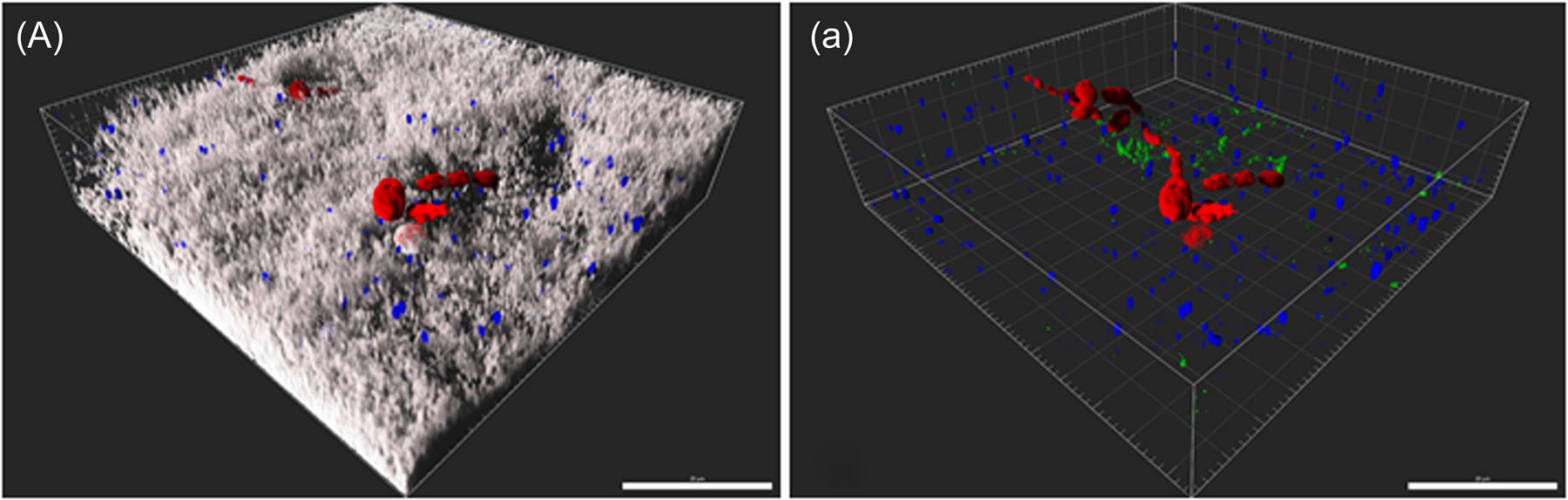
Confocal laser scanning microscopy (CLSM) images exhibiting maximum intensity projection (MIP) representations. (A, a) show an example of filamentous forms found in a six‐species biofilm (basic biofilm with *Streptococcus mutans* and *Streptococcus gordonii*). The scan of the filamentous forms was focused on *Candidia albicans*, and thus a 3.2× zoom was used for image acquisition and, to derive clearer images, the biofilms were scanned using the Shadow Projector Modus. The biofilms were stained with fluorescence in situ hybridization at 25% formamide; *C. albicans* (red), *S. mutans* (blue), and *S. gordonii* (green). In (A) the total biofilm mass is visualized with SYTO40 (light gray) compared to (a), where only *C. albicans*, *S. mutans*, and *S. gordonii* are visualized. The biofilm base in the three‐dimensional reconstructions is downward and the growth direction is upward; scale bar: 20 μm.

**Figure 9:**
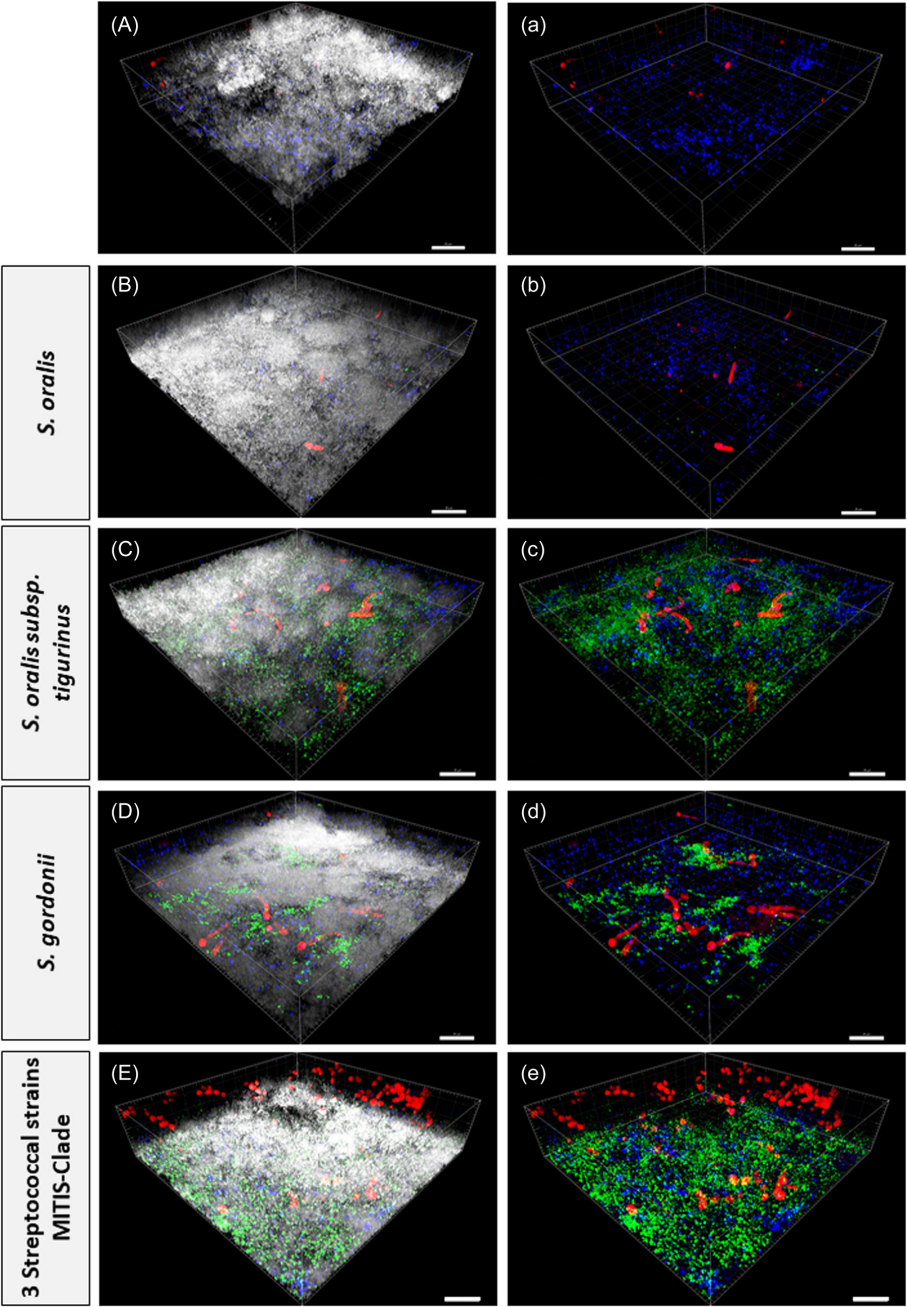
Confocal laser scanning microscopy (CLSM) images exhibiting three‐dimensional (3D) projection representations of biofilms with *Streptococcus mutans*. Due to the fluorescence in situ hybridization staining at 25% formamide with 16S ribosomal RNA probes (Caal1249‐Cy3, MUT590‐Cy5, and MIT447‐FAM), *Candida albicans* is visualized in red, *S mutans* in blue, and *Streptococcus oralis*, *S. oralis* subsp. *tigurinus* and *Streptococcus gordonii* in green. In (A–E), the total biofilm mass is visualized with SYTO40 (light gray) compared to (a–e), where only *C. albicans*, *S. mutans*, *S. oralis*, *S. oralis* subsp. *tigurinus* and *S. gordonii* are visualized. The biofilm base in the 3D reconstructions is downward and the growth direction is upward; scale bar: 20 μm.

The morphology of *C. albicans* was also studied. In the absence of *S. mutans, C. albicans* seemed to grow in its yeast form (Figure 7). However, when colonized together with streptococci of the mutans group and MSG, the formation of filamentous forms could be observed (Figure 8). Filamentous forms seemed to be more prominent in the presence of *S. gordonii* (no other additional MSG) combined with *S. mutans* (Figure 9Dd). Figure 8 shows an example of filamentous forms that were observed in this group. In confocal images of the basic biofilm as well as biofilms 1–8, the mass of *C. albicans* was determined using Imaris. However, it was found that the differences were not significant in any case, which is most likely due to the large variations.

## 4 DISCUSSION

The oral cavity harbors hundreds of different species of microorganisms, including both fungi and bacteria (Marsh & Zaura, 2017). The interactions between these different microorganisms, especially the cross‐kingdom interactions of streptococci and *C. albicans*, as well as their influence on oral diseases, have been of increasing interest worldwide, as they are associated with oropharyngeal diseases and the increased severity of dental caries (Allison et al., 2016; Diaz et al., 2012).

The present study used an in vitro multispecies “supragingival” biofilm model to analyze the interaction between members of the MSG and mutans streptococcal group and *C. albicans*. The model has been described previously and is reproducible and reliable (Thurnheer et al., 2014; Thurnheer & Belibasakis, 2015; Thurnheer & Paqué, 2021). In contrast to this biofilm model, saliva sampling is closer to real conditions in the oral cavity, especially regarding the proportions and numbers of different microorganisms that occur. The use of salivary or in situ biofilms also has an advantage over the application of in vitro biofilm models as there is a better adaption of the species in the inoculum. However, reproducibility is not given since different saliva donors are used (Sousa et al., 2016). The results of the present study indicate that the more streptococcal species are present in the biofilm, the more the CFU of *C. albicans* is reduced corresponding to the results described by Xu, Jenkinson et al. (2014). In the analyzed biofilms, the presence of *S. gordonii* resulted in the most significant reduction of *C. albicans* counts (Figure 2–5). However, Figure 5c shows no significant difference in *C. albicans* CFUs when *S. gordonii* was combined with *S. mutans* and other MSG (biofilms 7 and 8) or by itself (biofilm 4).

The fungal pathogen *C. albicans* is not normally found on the tooth surfaces of healthy and caries‐free people. At low pH values, it grows in its yeast form. However, survival at even lower pH levels (<4.5) is possible upon colonization with streptococci. In addition, the H_2_O_2_ production from the streptococci promotes hyphal formation by causing oxidative stress (Jenkinson et al., 1990; Nasution et al., 2008). The hyphal morphology of *C. albicans* is known to be its most resistant and pathogenic form (O'Donnell et al., 2015). Although *C. albicans* and *S. mutans* do not usually interact, enhancement of the interaction between them is achieved by a sucrose‐rich diet of the host (Falsetta et al., 2014). Whereas *Candida* species are not able to efficiently metabolize sucrose, *S. mutans* can (Williamson et al., 1993). However, the ability of *S. mutans* to process sucrose, thereby releasing fructose and glucose, considerably triggers the growth of *Candida* species, which contrasts our results (see above), suggesting that there may be other factors influencing the growth of *C. albicans*. Interestingly, the hyphal form of *C. albicans* can also be induced by an enhanced fructose metabolism (Han et al., 2011; Linke & Chang, 1976). Furthermore, when proximity to *C. albicans* exists, GtfB is secreted by *S. mutans*, which actively binds to the surface of *C. albicans*. In the process, a considerable quantity of α‐glucans (EPS) is formed from sucrose on the *C. albicans* surface. EPS provides binding sites for *S. mutans*, thereby allowing *C. albicans* to colonize the tooth surface (Falsetta et al., 2014). When N‐ or O‐linked mannans (outer layer of the fungal cell wall) are missing due to mutation, significantly reduced GtfB binding could be observed, resulting in a poorly developed mixed‐species biofilm and reduced EPS. Similarly, *S. mutans* that were defective in GtfB did not form mixed‐species biofilms with *C. albicans* (Hwang et al., 2017). Furthermore, Kim et al. (2017) demonstrated that the stimulation of bacterial growth in biofilms was triggered by extracellular factors resulting from interactions between *C. albicans* and *S. mutans*. While the findings from these studies highlight the fact that the influence of the biofilm matrix on the interactions between different microorganisms must not be ignored (Flemming et al., 2023), this aspect was not dealt with in the present work, although it may be the subject of future studies.

The hyphal formation is needed for biofilm formation by *C. albicans*, which can be induced by human saliva and *S. gordonii* (Bamford et al., 2009). The capacity to transition from the yeast growth form to the filamentous (hyphal) growth form is connected to both biofilm formation and virulence (Gow et al., 2002). Bamford et al. (2009) show an enhanced biofilm growth of *S. gordonii* and *C. albicans* when together in a mixed‐species biofilm. But since there is no method to measure the biofilm contributed by each component, the results are less revealing. On the other hand, complex physical and chemical interactions between *S. gordonii* and *C. albicans* promote a synergy in the creation of biofilms, which is evidenced by increased biomass and higher hyphal production. These interactions in the oral cavity may be important for encouraging a more rapid biofilm growth on denture surfaces or a higher hyphal penetration rate (Koo et al., 2018).

In vivo, research has demonstrated the impact of the synergy between *C. albicans* and MGS on the interaction between host and pathogen, with the growth of mixed biofilms (with *S. oralis*) encouraging neutrophil infiltration and increasing the severity of soft tissue lesions (Xu, Sobue, et al., 2014; Xu et al., 2016). This significantly differs from single‐species *C. albicans* biofilms, which are known to inhibit the neutrophil influx. Although cross‐kingdom synergies play a role in the pathogenesis of dental illnesses, interactions between the component species can also inhibit their capacity to control biofilm formation, growth, community alterations, and spatial structure (Bowen et al., 2018). Some metabolites from *S. mutans*, for instance, inhibit the hyphae formation of *C. albicans* (Joyner et al., 2010; Vílchez et al., 2010). Falsetta et al. (2014) also added the generated acidic environment of *S. mutans* that inhibits hyphae, as an explanation why yeast forms are found with *S. mutans* in the deeper layers of mixed biofilms. Furthermore, disruption of the hyphal formation of *C. albicans* is achieved by peptides secreted by *S. mutans* (Jarosz et al., 2009) and *S. gordonii* (Jack et al., 2015).

*S. mutans* and *S. gordonii* were shown to induce hyphal growth of *C. albicans* and to enhance the development of the biofilm (Bamford et al., 2009), as also mentioned by Falsetta et al. (2014) and Pereira‐Cenci et al. (2008). This study analyzed the changes in the morphology of *C. albicans* by using CLSM. Interestingly, *C. albicans* seemed to appear only as filamentous forms when one species of MSG was present in addition to *S. mutans*. In combination with several *Streptococcus* species, they again seemed to grow in their yeast form (Figure 9Ee). This could be explained by the increased competition between the different streptococci, which allowed *C. albicans* to grow in their less resistant yeast form. Upon filamentation, the correlation between CFU and candidal biomass is lost. As such, for example, while CFU levels may diminish in the presence of *S. gordonii*, overall levels of *C. albicans* biomass may be much higher due to hyphae formation. To account for such possibilities, the levels of *C. albicans* biomass for each biofilm combination were determined in the confocal images. However, no significant differences were found probably due to large variations in the different confocal images.

However, the co‐cultivation of *S. mutans* with *S. oralis* also induced a significant increase in biofilm production by *S. oralis* compared to what was observed in monospecies biofilms (Wen et al., 2010). Regarding the total CFU counts of groups containing *S. oralis*, the highest counts were found in the six‐species Zürich “supragingival” biofilm model (Figure 3), thereby supporting the theory of *S. oralis* being part of the normal oral microbiota. However, in a six‐species supragingival biofilm, Thurnheer and Belibasakis (2018) showed an overgrowth of *S. mutans* in the absence of *S. oralis*, suggesting competitive behavior in terms of growth and space. In this way, *S. oralis* is assumed to play the role of a homeostasis‐keeper and thus prevents a cariogenic shift of the biotic equilibrium. However, this overgrowth in the absence of *S. oralis* was not confirmed in the present investigation (Figure 2), which may be due to different growth conditions and composition of the biofilms described in these studies.

In summary, bacterial–fungal biofilms associated with oral diseases are of increasing significance and interest. More importantly, it has been suggested that shifts of oral bacteria to distant sites may even lead to systemic diseases such as coronary heart disease (Kuboniwa et al., 2012). This motivates the discovery of the detailed mechanisms of biofilm development, as it does not only concern the oral but also the overall health. In conclusion, the present study provides insights into the interactions between *C. albicans* and oral streptococci in biofilm models, which may infer the role of *C. albicans* in pathogenesis in the oral cavity. Our study also provides indications that *S. mutans* and *S. gordonii* induce the formation of filamentous forms of *C. albicans*, which may support their influence in reducing *C. albicans* counts. However, the number of filamentous forms was not in the foreground in this investigation and a deeper understanding of cross‐kingdom interaction, adhesion, signal pathways, and the impact of fungal pathogens in multispecies biofilms is required and should be the focus of future work.

## AUTHOR CONTRIBUTIONS

**Tenzin Kunchok Lueyar**: Formal analysis (lead); investigation (lead); validation (lead); visualization (lead); writing—original draft (lead); writing—review and editing (supporting). **Lamprini Karygianni**: Conceptualization (equal); methodology (equal); supervision (supporting); validation (supporting); writing—review and editing (equal). **Thomas Attin**: Project administration (lead); supervision (supporting); writing—review and editing (supporting). **Thomas Thurnheer**: Conceptualization (equal); methodology (equal); supervision (lead); validation (supporting); writing—review and editing (equal).

## CONFLICT OF INTEREST STATEMENT

None declared.

## ETHICS STATEMENT

All procedures performed in the study were in accordance with the ethical standards of the institutional and/or national research committee and with the 1964 Declaration of Helsinki and its later amendments or comparable ethical standards. No approval is required by the Ethics Committee of the University of Zurich in case pooled saliva is used in the framework of in vitro assays in the lab.

## Data Availability

All data are provided in this article.
